# Using Social Media to Facilitate Communication About Women’s Testing: Tool Validation Study

**DOI:** 10.2196/35035

**Published:** 2022-09-26

**Authors:** Tara Coffin, Deborah Bowen, Karen Lu, Elizabeth M Swisher, Nadine Rayes, Barbara Norquist, Stephanie V Blank, Douglas A Levine, Jamie Nadine Bakkum-Gamez, Gini F Fleming, Olufunmilayo I Olopade, Iris Romero, Alan D'Andrea, Denise R Nebgen, Christine Peterson, Mark F Munsell, Kathleen Gavin, Jamie Crase, Deborah Polinsky, Rebecca Lechner

**Affiliations:** 1 University of Washington Seattle, WA United States; 2 MD Anderson Cancer Center Houston, TX United States; 3 Mount Sinai Medical Center New York, NY United States; 4 New York University Langone Health New York, NY United States; 5 Mayo Clinic Rochester, MN United States; 6 University of Chicago Chicago, IL United States; 7 Dana-Farber Cancer Institute Boston, MA United States; 8 Minnesota Ovarian Cancer Alliance Minneapolis, MN United States; 9 SHARE New York, NY United States

**Keywords:** ovarian cancer, hereditary cancer, genetic testing, online social media recruitment, Facebook, social media, mobile phone

## Abstract

**Background:**

Strong participant recruitment practices are critical to public health research but are difficult to achieve. Traditional recruitment practices are often time consuming, costly, and fail to adequately target difficult-to-reach populations. Social media platforms such as Facebook are well-positioned to address this area of need, enabling researchers to leverage existing social networks and deliver targeted information. The MAGENTA (Making Genetic Testing Accessible) study aimed to improve the availability of genetic testing for hereditary cancer susceptibility in at-risk individuals through the use of a web-based communication system along with social media advertisements to improve reach.

**Objective:**

This paper is aimed to evaluate the effectiveness of Facebook as an outreach tool for targeting women aged ≥30 years for recruitment in the MAGENTA study.

**Methods:**

We designed and implemented paid and unpaid social media posts with ongoing assessment as a primary means of research participant recruitment in collaboration with patient advocates. Facebook analytics were used to assess the effectiveness of paid and unpaid outreach efforts.

**Results:**

Over the course of the reported recruitment period, Facebook materials had a reach of 407,769 people and 57,248 (14.04%) instances of engagement, indicating that approximately 14.04% of people who saw information about the study on Facebook engaged with the content. Paid advertisements had a total reach of 373,682. Among those reached, just <15% (54,117/373,682, 14.48%) engaged with the page content. Unpaid posts published on the MAGENTA Facebook page resulted in a total of 34,087 reach and 3131 instances of engagement, indicating that around 9.19% (3131/34,087) of people who saw unpaid posts engaged. Women aged ≥65 years reported the best response rate, with approximately 43.95% (15,124/34,410) of reaches translating to engagement. Among the participants who completed the eligibility questionnaire, 27.44% (3837/13,983) had heard about the study through social media or another webpage.

**Conclusions:**

Facebook is a useful way of enhancing clinical trial recruitment of women aged ≥30 years who have a potentially increased risk for ovarian cancer by promoting news stories over social media, collaborating with patient advocacy groups, and running paid and unpaid campaigns.

**Trial Registration:**

ClinicalTrials.gov NCT02993068; https://clinicaltrials.gov/ct2/show/NCT02993068

## Introduction

### Background

High participant response rates and recruitment yields are critical to public health research but are difficult to achieve [1-3]. Traditional recruitment practices, including radio or newspaper advertising, in-person referrals, and flyers, are often time consuming to implement, costly, and fail to adequately target difficult-to-reach populations [4,5]. The initial net cast using these types of recruitment methods may result in a high number of interested parties; however, such efforts result in proportionately fewer eligible and enrolled participants, and certain demographics are frequently left underrepresented [6]. Social media is well-positioned to address many of these issues and improve participant recruitment by providing new platforms for people to learn about public health research [7-10].

The term *social media* broadly describes a variety of web-based social networking platforms or web-based spaces where the public can generate, engage with, and share information, including platforms such as Facebook, Twitter, and Instagram [11,12]. Social media enables researchers to deliver information to a wide audience; target specific groups of people, including hard-to-reach subpopulations; and adapt outreach efforts on an ongoing basis [7-10]. Current research indicates that social media recruitment methods are an improvement over traditional methods in terms of both cost and effectiveness [13-16].

Facebook, used by more than three-quarters of adults on the web, is particularly well-suited for research recruitment [17,18]. Over Facebook, users can engage with user-generated content, publish photos on their Facebook pages, post status updates, and share information with friends and family. Users follow the content of interest and engage socially with paid advertisements and other content. Researchers can leverage this environment, creating content tailored for specific populations using online behavioral advertising (OBA) and respondent-driven sampling to improve reach [19].

OBA data can help researchers improve their marketing reach. OBA data include information collected from a broad range of web-based sources about the behaviors that users exhibit on the web [20]. OBA appeals to researchers in public health, seeking to improve recruitment tools and offering an alternative outreach method with a broader reach that may overcome certain recruitment barriers, such as geographic limitations [21-25]. Instead of wondering whether a flyer is posted in the right place for the right type of individual to see, researchers can guarantee that their message is being displayed to the intended person. This approach is not without its limitations, and some health professionals and researchers have expressed reluctance, citing concerns about biased sampling or reach that may accompany social media platforms [26] and privacy [12,27,28].

Facebook also allows public health professionals to leverage existing social networks through snowball sampling [29,30]. Snowball sampling, which has traditionally taken place offline, can capitalize on existing web-based social networks, such as patient advocacy groups [30,31]. By encouraging a small sample of a target population to refer others to a research study, snowball sampling helps researchers access hidden subpopulations that are typically difficult to sample using traditional recruitment methods [30]. From snowball sampling to inviting opportunities to shape the tone, imagery, and content to fit the needs of the intended audience, social media is well-positioned to function as a targeted communication tool. With these advantages, social media has the potential to take traditional snowball sampling one step further, enabling researchers to potentially connect with harder-to-reach populations [32]. This quality grants social media recruitment the ability to potentially shift the pattern of health inequities, improving the representation of certain communities in the research arena [33].

Recent reviews indicate that most studies using Facebook to recruit participants for health research have focused on people aged 18 to 30 years [8,34]. In comparison, few studies have evaluated social media as a means of recruiting people affected by cancer who are aged ≥35 years [34], and no studies have explored how social media recruitment performs when targeting women at risk for ovarian cancer. The consensus is that older people may be less likely to adopt new technologies, such as social media [34,35]. Other studies have reported high reach but low engagement among social media users, resulting in a high attrition rate for social media recruitment [36]. However, this research failed to examine advertisement content or take the growth of the social media platform into consideration. As the social media base continues to grow, the profile of the average user evolves, and with it, the age of the average Facebook user continues to increase [37]. With this evolution in mind, ongoing assessment is needed to evaluate the effectiveness of social media for research participant recruitment across different demographics, and more research is needed to better understand how Facebook functions as a recruitment tool in the context of ovarian cancer [20].

### Study Aims

This research sought to determine whether Facebook is an effective recruitment tool for targeting women aged ≥30 years for recruitment into the MAGENTA (Making Genetic Testing Accessible) study by evaluating innovative methods for the recruitment of research participants using Facebook. To accomplish this objective, a series of posts and advertisements, including paid and unpaid posts, were published and assessed on an ongoing basis. These materials used a variety of imagery and languages and leveraged Facebook’s OBA tools to target specific populations and eligible participants. We hypothesized that unpaid Facebook posts and Facebook advertisements would improve the reach of the study material and result in improved study enrollment.

## Methods

### About the MAGENTA Study

The MAGENTA study was a nationwide Stand Up To Cancer initiative that sought to improve access to genetic testing for ovarian cancer. The study recruited and randomized 3839 women from the United States with a potentially increased risk of ovarian cancer. Participants were randomized to 1 of 4 arms, receiving a combination of pretest or posttest telephone genetic counseling and pretest or posttest web-based education with optional telephone counseling [38]. The active recruitment period took place between April 2017 and January 2020. This study received institutional review board approval from the MD Anderson Cancer Center and was a collaborative effort that included several cancer research centers and patient advocacy groups, including the Ovarian Cancer Research Alliance, National Ovarian Cancer Coalition, and Minnesota Ovarian Cancer Alliance.

**Figure 1:**
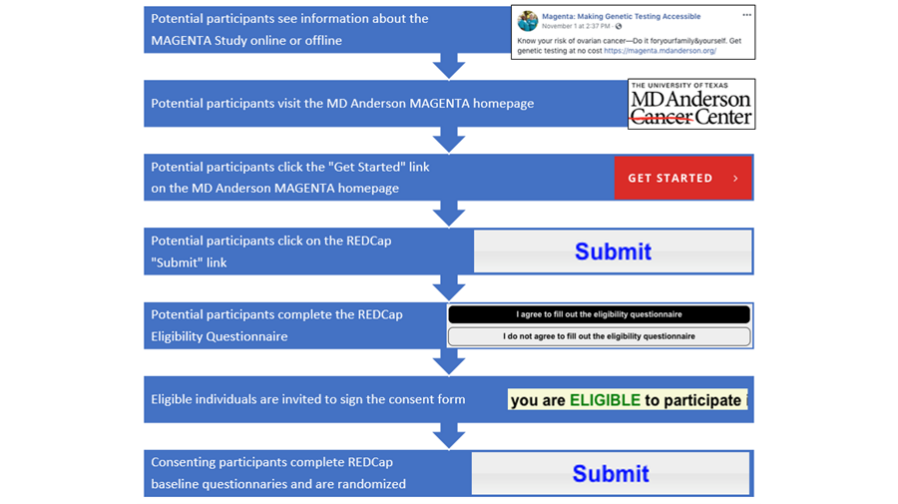
Illustration of the web-based communication system used by the MAGENTA (Making Genetic Testing Accessible) study. REDCap: Research Electronic Data Capture.

Once potential participants had learned about the MAGENTA study, they were prompted to visit the study website. From there, interested parties clicked to participate in the web-based communication system, starting with study information and then moving through the eligibility screen, informed consent, and enrollment (Figure 1). Data were collected at baseline and follow-up using REDCap (Research Electronic Data Capture), an electronic survey tool sponsored by the University of Washington (WA). All outreach materials received institutional review board review through the MD Anderson Cancer Center. The results of the MAGENTA study indicate that electronic genetic education and results released without genetic counseling were noninferior with regard to patient distress. Importantly, research also found that providing genetic education and results in this capacity was associated with higher test completion and lower distress [38].

### Developing a Media Kit

Adapting the methods outlined by Carter-Harris et al [39] and Musiat et al [40], the study media kit was developed in collaboration with key stakeholders. This group comprised health care professionals from cancer care and research centers and patient advocates from advocacy groups across the United States, including those listed previously. Patient advocates were consulted extensively during the development of the study materials, including the media kit described in the following sections. The media kit included Facebook recruitment materials and a list of social media contacts, such as patient advocacy groups and other groups with an apparent interest in breast and ovarian cancer.

**Figure 2:**
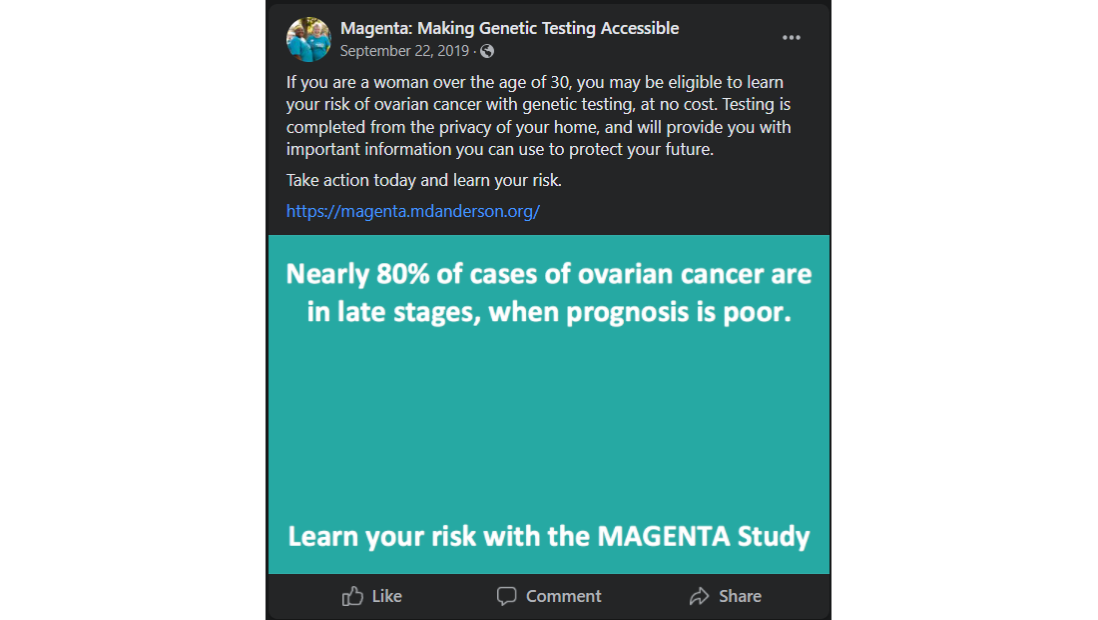
An example Facebook post containing a still image, study link, and brief description of the outreach. This type of post was used in both for unpaid posting and paid advertising campaigns.

**Figure 3:**
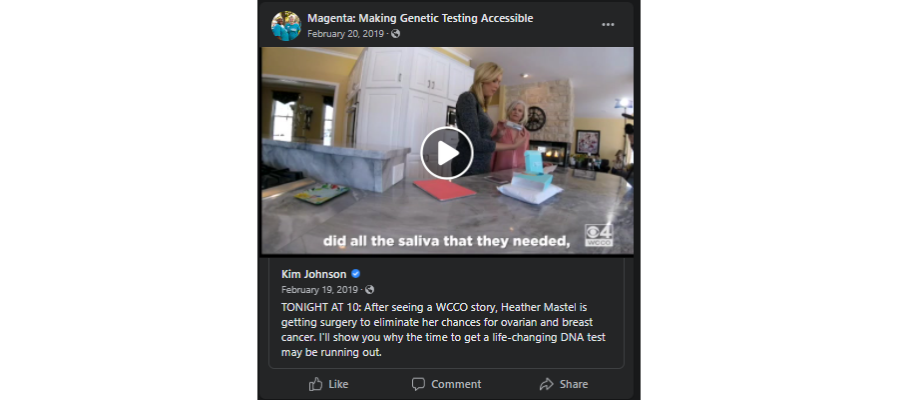
An example of a Facebook post sharing the WCCO news story, which includes a video of the news story and a brief text section. This type of post is an example of a boosted post that was used for unpaid posting.

**Figure 4:**
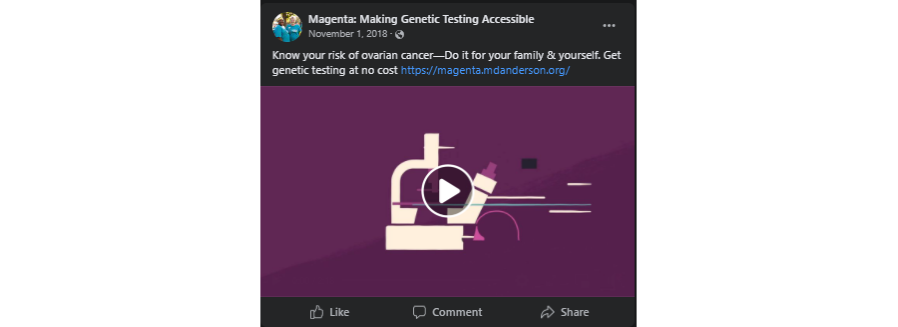
An example of a Facebook post containing the study video, study link, and a brief description of the outreach. This type of post was used in both unpaid and paid advertising campaigns.

The media kit also included different types of posts generated for recruitment purposes, including paid advertisements, unpaid posts, sample tweets, a list of relevant hashtags to incorporate into posts, and a selection of media for use across all social media posts and advertisements (example posts can be reviewed in Figures 2-4). Unpaid Facebook posts and paid advertisements included at least one media component, a brief description of the study, relevant hashtags, and a link to the study home page (Figure 1). A MAGENTA Facebook page was created to develop trust with potential participants [41,42]. The Facebook page provided basic information about the study, served as a platform for sharing unpaid and paid social media posts, and directed potential participants to the study website. Materials from the media kit were assessed by patient advocates and underwent usability testing. Advertisements and posts were created with tone and imagery in mind, focusing on content related to ovarian cancer research that elicited a combination of the following concepts adapted from Batterham [43]:

1. Content instills a sense of collaboration, conveying the idea that one is participating in research as a member of a team to address a health problem (in this case, ovarian cancer was framed as the problem).
2. Content instills a sense of independence or conveys the idea that one is addressing the problem of ovarian cancer as an individual through research participation.
3. Content instills a sense of altruism, conveying the idea that the individual is participating in research for the benefit of others.
4. Content instills a sense of self-gain or self-preservation, conveying the idea that the individual is participating in research for personal gain.

### Publishing Paid Advertisements and Unpaid Posts

Unpaid posts were published directly on the MAGENTA Facebook page on a regular basis and on patient advocacy Facebook pages. Paid advertisements were published using Facebook’s advertising tool. Once the objective or goal of the campaign (eg, post engagement, website clicks, and video views) was set, the audience was identified using Facebook’s audience-targeting tool. Targeted populations for the purposes of this study included English-speaking women ≥aged 30 years living in the United States. Additional geographic and behavioral targeting was included on a case-by-case basis and is described in greater detail in Table 1.

Census data were used to inform additional geographic and socioeconomic targeting and included data surrounding racial-ethnic groups and the rurality of the location. These variables were layered using ArcGIS Pro (version 2.5; Esri) to select the specific geographic targets. ArcGIS is a mapping and analysis tool that allows users to use a geographic information system to capture, manipulate, and analyze geospatial data. Once the audience was selected, advertising content was uploaded to Facebook, a campaign budget was selected, and a campaign schedule was set. On the basis of the intended audience, Facebook uses OBA approaches to push out content with the above parameters in mind. Although targeting affects results, over social media platforms, including Facebook, the budget arguably has the most impact on reach, and larger budgets are generally associated with more results, assuming that appropriate targeting is used.

**Table 1:** Description of Facebook paid campaign content and audience.

Advertisement name	Advertisement description				Audience description	
	Objective	Media	Age (years)	Geographic		Interests or additional targeting
Lookalike Audience Campaign	Conversions	Video	30 to ≥65	United States		Lookalike (United States, 1%)—website traffic
CA^a^ Campaign 1	Conversions	Image	30 to 55	Salinas, CA		Breast cancer awareness, National Breast Cancer Foundation, and National Ovarian Cancer Coalition
CA Campaign 2	Conversions	Video	30 to ≥65	Salinas, CA		Advocacy groups for patients with multiple hereditary cancers, focusing on ovarian and breast cancer
CA Campaign 3	Conversions	Image	30 to 60	Downey, Pomona, Santa Ana, CA		Breast cancer awareness and Telemundo or Univision
Cascade Testing 1	Conversions	Image	30 to 55	United States		Facing Our Risk of Cancer Empowered
Cascade Testing 2	Conversions	Image	30 to 55	United States		Facing Our Risk of Cancer Empowered
African American Target Campaign	Traffic	Video	30 to ≥65	Birmingham, Montgomery, Alabama; Miami Gardens, Florida; Savannah, Georgia; New Orleans, Los Angeles; Baltimore, Maryland; Detroit, Flint, MI; Jackson, Mississippi; Memphis, Tennessee		N/A^b^
NY^c^ Campaign	Traffic	Video	30 to ≥65	New York state		Advocacy groups for patients with multiple hereditary cancers, focusing on ovarian and breast cancer
BCOC^d^ Interest Campaign	Traffic	Image	30 to ≥65	United States		N/A
BCOC Interest Campaign	Engagement	Image	30 to ≥65	Seattle, WA^e^		The Breast Cancer Research Foundation, national breast cancer Awareness Month, Susan G Komen for the Cure or Living Beyond Breast Cancer
BCOC Interest Campaign	Traffic	Image	30 to ≥65	United States		N/A
BCOC Interest Campaign	Traffic	Study video	30 to 50	United States		Advocacy groups for patients with multiple hereditary cancers, focusing on ovarian and breast cancer; keywords related to breast and ovarian cancer
BCOC Interest Campaign	Traffic	Study video	30 to 50	United States		Advocacy groups for patients with multiple hereditary cancers, focusing on ovarian and breast cancer; keywords related to breast and ovarian cancer
BCOC Interest Campaign	Traffic	Study video	30 to 50	United States		Advocacy groups for patients with multiple hereditary cancers, focusing on ovarian and breast cancer; keywords related to breast and ovarian cancer
Latino Target Campaign	Traffic	Image	30 to ≥65	Brownsville, El Paso, Laredo, McAllen, Texas; Hialeah, Florida; Downey, Oxnard, Pomona, Salinas, Santa Ana, CA		N/A
BCOC Interest Campaign	Traffic	Image	30 to ≥65	United States		Advocacy groups for patients with multiple hereditary cancers, focusing on ovarian and breast cancer; keywords related to breast and ovarian cancer
BCOC Interest Campaign	Traffic	Study video	30 to ≥65	United States		Advocacy groups for patients with multiple hereditary cancers, focusing on ovarian and breast cancer; keywords related to breast and ovarian cancer
WA Campaign	Conversions	Image	30 to 50	Seattle, WA		Essence (magazine) and Latina (magazine)

^a^CA: California.

^b^N/A: not applicable.

^c^NY: New York.

^d^BCOC: breast cancer and ovarian cancer.

^e^WA: Washington.

### Other Recruitment Efforts

All Facebook recruitment efforts took place alongside traditional recruitment efforts as part of the MAGENTA study. Traditional recruitment efforts included clinician referrals, direct emails from patient advocacy groups, the dissemination of study information at provider and patient advocate conferences, and sharing physical flyers in patient advocacy and clinical settings. Traditional efforts were largely based on the participating cancer research institutes, organizations, and patient advocacy groups. Study recruitment commenced with traditional methods, allowing for a controlled launch that allowed for an additional real-time usability assessment of the web-based communication system. In this first round of recruitment, enrollment relied primarily on word of mouth and flyer dissemination, both of which were facilitated by collaborating with patient advocacy groups. Following this controlled outreach, the study team expanded the outreach to include social media posts, as described above, in an effort to expand the reach of messaging.

### Evaluating Paid Advertisements and Unpaid Posts

Facebook analytics captured how users interacted with the social media MAGENTA content. Analytics included, among others, the following: *engagement* is defined as any time an individual takes action on the post, where action includes a click, comment, share, or view; *results* are defined as the number of times an advertisement achieved a specific outcome, delineated by the campaign objective; *reach* is defined as the number of people who saw the advertisement at least once; *impressions* are the number of times an advertisement was on a screen; *clicks* refer to the number of times someone clicked on the advertisement; and *video plays*. The study team also reviewed the *cost per result*. *Cost per result* was calculated by dividing the total amount of money spent by the number of *results*, which may include the number of video views or website visits, for example, obtained over the course of the campaign. Analytics, including cost, were reviewed daily to assess the effectiveness and provide opportunities to adjust campaign content or targeting. The same information was collected for unpaid posts published directly on the MAGENTA study’s Facebook page. If at any time the MAGENTA study website or another part of the web-based communication system became overburdened, advertisements were pulled, or turned off, until the traffic subsided.

### Ethics Approval

This study, including all outreach materials, received institutional review board review through MD Anderson Cancer Center (2016-0298).

## Results

### Overview

**Figure 5:**
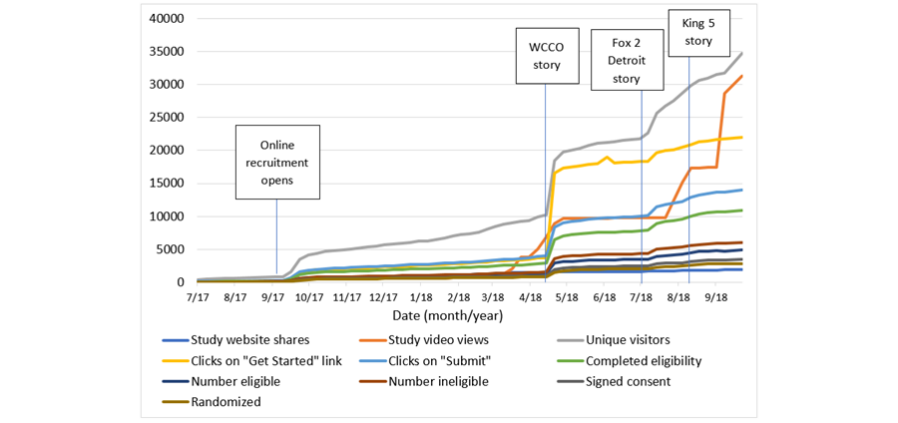
Timeline of enrollment trends and recruitment events captured during the active social media recruitment period (September 2017 to October 2018) and the number of responses received at different steps in recruitment activity.

Active social media recruitment for the MAGENTA study took place between September 2017 and October 2018. The MAGENTA study relied on traditional recruitment methods starting in April 2017 until September 2017. Traditional recruitment methods continued throughout the social media recruitment period; however, the study team focused on web-based recruitment efforts in the interest of improving the reach across all 50 states. The recruitment timeline can be viewed in Figure 5. During the active social media recruitment period, Facebook materials reached a total of 407,769 users, generating 57,248 (14.04%) instances of engagement, suggesting that approximately 14.04% of people who saw information about the MAGENTA study on Facebook engaged with the content. These numbers did not identify unique users and excluded posts published on Facebook pages managed by other breast and ovarian cancer groups. During this time, the MAGENTA study home page was shared 1948 times, and the MAGENTA study video was viewed 31,358 times (Table 2).

**Table 2:** Summary of enrollment and randomization data.

Step in enrollment protocol			Total unique visitors
**MAGENTA^a^** **Facebook page content, n**			
	Reach	407,769	
	Engagement (clicks, reactions, comments, and Facebook content shares)	57,248	
	Study website shares	1948	
	Study video views	31,358	
**MAGENTA MD Anderson webpage**			
	Unique visitors, N	34,715	
	Clicks on *Get Started* link, n (%)	22,029 (63.5)	
**REDCap^b^ introductory message, n (%)**			
	Clicks on *Submit* link	14,025 (40.4)	
**REDCap eligibility** **, n (%)**			
	Started eligibility questionnaire	13,983 (40.3)	
	Completed eligibility questionnaire	10,883 (31.3)	
	Number eligible	4887 (14.1)	
	Number ineligible	5996 (17.3)	

^a^MAGENTA: Making Genetic Testing Accessible.

^b^REDCap: Research Electronic Data Capture.

### Users Learn About the MAGENTA Study Over Television and Social Media

Of the 13,983 respondents to the MAGENTA REDCap eligibility questionnaire during the active social media recruitment period, <1% (n=23, 0.16%) indicated that they learned about the study from a magazine, <1% (n=86, 0.62%) from the radio, <2% (n=253, 1.81%) from a health care provider, >3% (n=459, 3.28%) from a patient advocacy group, and <8% (n=1102, 7.88%) from a friend. Approximately 8.64% (1209/13,983) indicated that they learned about the study from a family member, whereas 27.44% (3837/13,983) indicated that they learned about the study on the web, either from social media or another webpage, and 28.16% (3938/13,983) from television. Among those who reported that they learned about the study from the internet, 16.94% (2369/13,983) specifically cited social media. A total of 21.7% (3034/13,983) of individuals who responded to the REDCap eligibility questionnaire did not indicate where they had heard about the study. Some respondents reported learning about the study from more than one source.

### Social Media Response

Paid advertisements (Table 3) reported a total reach of 373,682 during the active social media recruitment period. Among those reached, 3.57% (13,357/373,682) clicked on a link and 14.48% (54,117/373,682) engaged with the page content. Paid campaigns also generated 19,792 video plays and 9095 website conversions, which were defined as instances where a potential participant viewed the page content on the MAGENTA home page. Paid advertisements using the study video resulted in a total reach of 54,992 and 28,586 instances of page engagement. Post promotions, or paid advertisements that focused on increasing the reach of a post, resulted in 2666 reach and 97 instances of engagement. Conversion campaigns resulted in 268,052 reaches, 35,904 instances of engagement, and 9095 conversions. Campaigns seeking to drive traffic to the MAGENTA study website resulted in 80,120 reaches, 18,158 instances of engagement, and 1697 times that a unique user clicked on the link to the MAGENTA home page.

Almost all users engaged with paid advertisements from a handheld mobile device, such as a smartphone or tablet, rather than through a desktop computer. Most users engaged with paid advertisements from their Android device (35,806/373,682, 9.58%), followed by iOS devices (16,466/373,682, 4.41%) (eg, iPad). Approximately 9.98% (26,752/268,168) of the women aged <54 years reached by the advertisement content engaged with the advertisement, whereas approximately 26.62% (12,226/45,930) of women aged between 55 and 64 years who saw the paid content engaged, and 43.95% (15,124/34,410) of women aged ≥65 years who saw MAGENTA advertisements engaged with advertisement content. The difference observed between the above age demographics regarding reach to engagement was statistically significant (*P*<.001). Unpaid posts published on the MAGENTA Facebook page resulted in 34,087 reaches and 3131 engagements. These numbers do not include social media posts published on other non-MAGENTA Facebook groups and pages.

**Table 3:** Global summary of results for all paid campaigns.

Advertisement name and objective	Reach	Post engagement, n (%)	Page engagement, n (%)	Video plays, n (%)	Link clicks, n (%)	Results, n (%)	Cost per results (US $)
BCOC^a^ Interest 8 and traffic	2280	43 (1.89)	46 (2.02)	N/A^b^	37 (1.62)	37 (1.62)	1.35
Latino 7 and traffic	11,062	217 (1.96)	223 (2.02)	N/A	204 (1.84)	204 (1.84)	1.23
BCOC Interest 6 and traffic	7536	1397 (18.54)	1397 (18.54)	N/A	266 (3.53)	266 (3.53)	0.94
BCOC Interest 5 and traffic	10,436	2069 (19.83)	2069 (19.83)	N/A	280 (2.68)	280 (2.68)	1.07
BCOC Interest 4 and traffic	34,648	6536 (18.86)	6536 (18.86)	N/A	640 (1.85)	540 (1.56)	1.94
BCOC Interest 9 and traffic	2050	351 (17.12)	351 (17.12)	N/A	20 (0.98)	20 (0.98)	2.00
African American and traffic	9382	7308 (77.89)	7308 (77.89)	N/A	319 (3.40)	319 (3.40)	0.63
Cascade Testing 1-2 and conversions	132,480	3341 (2.52)	3342 (2.52)	N/A	3326 (2.51)	2514 (1.90)	0.17
WA^c^ and conversions	20,732	313 (1.51)	317 (1.53)	N/A	298 (1.44)	73 (0.35)	5.29
CA^d^ 3 and conversions	35,976	2022 (5.62)	2022 (5.62)	N/A	2022 (5.62)	1692 (4.70)	0.21
NY^e^ Campaign and traffic	395	186 (47.09)	186 (47.09)	N/A	14 (3.54)	14 (3.54)	0.53
Lookalike 1 and conversions	19,240	7142 (37.12)	7142 (37.12)	12,244 (63.64)	1130 (5.87)	880 (4.57)	0.40
BCOC Interest 1 and traffic	1587	31 (1.95)	33 (2.08)	N/A	7 (0.44)	7 (0.44)	3.77
BCOC Interest 2 and engagement	334	55 (16.47)	55 (16.47)	N/A	N/A	55 (16.47)	0.27
BCOC Interest 3 and traffic	746	10 (1.34)	10 (1.34)	N/A	10 (1.34)	10 (1.34)	1.50
CA 2 and conversions	35,752	21,444 (59.98)	21,444 (59.98)	7548 (21.11)	3147 (8.80)	2530 (7.08)	0.29
CA 1 and conversions	23,872	1637 (6.86)	1637 (6.86)	N/A	1637 (6.86)	1406 (5.89)	0.35

^a^BCOC: breast cancer and ovarian cancer.

^b^N/A: not applicable.

^c^WA: Washington.

^d^CA: California.

^e^NY: New York.

### MAGENTA Study Enrollment and Randomization Summary

There were 34,715 unique visitors to the MD Anderson home page during the active social media recruitment period and 22,029 (63.46%) unique clicks. Approximately 63.46% (22,029/34,715) of users who visited the MD Anderson MAGENTA home page during this period clicked on the *Get Started* link, which directed them to the landing page on the REDCap system. The *Submit* button on the REDCap landing page received a total of 40.4% (14,025/34,715) of clicks, and the eligibility questionnaire on REDCap was completed 31.35% (10,883/34,715) of the times. Of the completed questionnaires, 14.02% (4887/34,715) were eligible. The enrollment and randomization data from the active social media recruitment period are summarized in Table 2.

### Social Media Campaigns and News Stories Influence Enrollment Response

The general recruitment activity following paid advertisements was tracked and compared with periods when paid advertisements were not running. Because of the overlap in campaigns and television news stories, changes in recruitment activity around paid campaigns were not reported for all campaigns, and in some cases, the observation period following the campaign was excluded because of another campaign running during that time. There was an uptake in the completed eligibility questionnaires following individual and successive paid campaigns. Before 2 paid advertisements, which ran back to back in November 2017, there was a rate of 5.2 eligibility questionnaires completed daily. This number increased to a rate of 6.9 during these campaigns and in the week following the campaign. During the 2 weeks before another pair of paid advertisements, published sequentially in March 2018, there was a rate of 7.4 completed eligibility questionnaires daily, increasing to a rate of 12.7 during and in the 2 weeks following the campaign.

Enrollment following paid advertisement campaigns with a narrow geographical focus was further assessed. These campaigns included a targeted campaign in WA State (WA Campaign) and a campaign with multiple advertisements in California (CA; CA Campaign 1, CA Campaign 2, and CA Campaign 3), as seen in Table 3. The WA Campaign reached 20,733 people, about 1.43% (298/20,733) of whom clicked on the webpage link, and 0.35% (73/20,733) went on to view content on the MD Anderson MAGENTA page. Throughout this campaign, a total of 32 individuals from WA State completed the eligibility questionnaire at a rate of 3.7 completed eligibility questionnaires per day. Before the social media campaign, a rate of 0.5 completed eligibility questionnaires daily.

The advertisement campaign targeting CA comprised 3 advertisements (CA Campaign 1, CA Campaign 2, and CA Campaign 3). This campaign had a reach of 95,600. Just >7% (6806/95,600, 7.12%) of these reaches resulted in a webpage link click, and some (5628/95,600, 5.89%) went on to view content on the MD Anderson MAGENTA page. During and after the immediate campaign, a total of 74 individuals from CA completed the eligibility questionnaire at a rate of 1.5 completed eligibility questionnaires per day. Before this campaign, there was a rate of 0.6 completed eligibility questionnaires per day from the state of CA.

During the active social media enrollment period, several television news stories, spearheaded by patient advocates and clinicians affiliated with the study, about the MAGENTA study were broadcast, including a story from WCCO based in Minnesota, [44], a Fox 2 Detroit story from Michigan, [45], and the King 5 story based in the WA State [46]. These news stories were widely shared over social media. In the month following the WCCO story, completed eligibility questionnaires from Minnesota increased from <0.5 per day to almost 123 per day. An increase in completed questionnaires was also observed following the release of the Fox 2 Detroit story. In the month immediately following this story, the number of completed eligibility questionnaires increased from 0.3 per day to 31 per day. Similarly, in the month following the King 5 story, completed eligibility questionnaires from WA State increased from 0.6 per day to 25 per day. These increases in enrollment and recruitment activities are shown in Figure 5. Other increases, specifically those observed in study video views, aligned with paid Facebook advertising campaigns, where *video views* was the campaign objective.

## Discussion

### Principal Findings

This study demonstrated that Facebook is a useful way of reaching women aged >30 years who have a potentially increased risk of ovarian cancer through paid advertising, unpaid social media posts, and promoting news stories on social media. The key learning points include the following:

1. Campaign objectives that require more participant action to reach the end
  result
  generate passive engagement along the way.
2. Multimedia posts, specifically those with a video, create opportunities for engagement.
3. Effective social media outreach requires close collaboration with patient advocacy groups.
4. Web-based behavioral advertising can support targeted message delivery but is limited to those present on a specific platform.

In addition to these lessons, this research highlights other important limitations of social media outreach. Each of these learning points is addressed in greater detail below in the following sections.

### Campaign Objectives That Require More Action Generate Passive Engagement

More than one-quarter of the participants filling out the eligibility survey had heard about the study through social media, and another 28.16% (3938/13,983) through traditional media sources (ie, television news) that were then amplified by social media. Targeted, regional, paid Facebook advertising resulted in measurable increases in relevant regional enrollment for approximately 2 weeks following each campaign. These recruitment sources were essential to the successful completion of MAGENTA enrollment and resulted in a wide national representation, with participants enrolling from all 50 states.

The engagement indicators reported across paid advertising were varied by the campaign (Table 3). The campaign objective, budget, schedule, duration, and targeted population all influenced the response rate and participant engagement. During the reported recruitment period, demographic targeting was modified by age, geographic location, and expressed interests on an ongoing basis. Campaigns that were more finely targeted by geographic location and prior engagement with cancer information or groups tended to cost more per result when compared with campaigns with broader targeting, presumably as the more customized population was comparatively smaller and more difficult to reach. Similarly, when the objective of the campaign required more action on the part of the participant to meet the objective, the cost per result increased. In other words, if the objective of the campaign was to get the participant to view the material on the study website, which would require the advertisement to appear on their screen, the participant must actually see the advertisement, click on the advertisement, go to the study home page, and spend a few moments with the study home page open on their browser. As a result, for example, this specific objective required a higher amount of engagement than a *post view* would. This also means that any advertisement with a multistep objective requiring more engagement accrued more upstream engagement. In the case of website views, to get a certain number of people to view the website, the Facebook advertising system required more people to see the initial post, spend time viewing that post, click the link, and so on. With this pattern in mind, we found that it was possible to increase post engagement upstream by focusing on downstream objectives that require more interaction to achieve. This incidental engagement also facilitated opportunities for repeated exposure, making it more likely for individual users to see information about the study more than once, potentially building brand recognition and familiarity.

### Multimedia Posts Create Opportunities for Engagement

Multimedia elements, such as the study video, were important for outreach during the study enrollment period. For example, Figure 5 depicts different ways that potential participants could interact with the web-based communication system, illustrating engagement with the study video, among other variables. The study video views fluctuated with the paid campaigns. Although many of the engagement increases observed in Figure 5 were connected to news stories and the subsequent boosting of these stories over social media, there were increases in study video views related to paid campaigns that had a video view objective. As we did not have a mechanism built into the web-based communication system that allowed us to determine how many participants learned about the study from watching the study video specifically, we were unable to calculate how many video views resulted in enrollment. Despite this limitation, video views likely helped build familiarity among the potential participants.

### Social Media Outreach Is Only as Strong as Your Relationships With Patient Advocates

This study also demonstrates the importance of including patient advocates as members of a multidisciplinary research team and using social media to boost patient advocate-spearheaded recruitment efforts. Patient advocacy groups supporting the MAGENTA study were critical to the success of the study. They not only helped facilitate televised and print news stories but also disseminated study information across their established web-based, as well as in-person, social networks. Importantly, patient advocates working with the study team also helped shape targeted advertising campaigns through Facebook’s campaign targeting tools, which helped identify and boost content for individuals who followed patient advocacy Facebook pages.

Patient advocates were instrumental in designing accessible recruitment materials, getting news stories published, and supporting story circulation. Following the release of news stories featuring the MAGENTA study, there was a consistent increase in enrollment trends, with 28.16% (3938/13,983) of potential participants reporting that they learned about the study over television, referencing specific news stations featuring news clips about the study. These news segments, spearheaded by patient advocates, played a central role in the study recruitment. Although these stories originated via traditional media, either as televised news stories or similar publications, social media still likely played a role in promoting this content. Over social media, more people were able to view and share the news stories, making these news features more accessible. The inclusion of multimedia content, such as videos, appeared to extend this reach further, making web-based content easier to view and share. The advantage of video media is well-documented, with other research confirming that videos and other media-rich posts perform better than text-based content alone [47,48].

Given the spikes in page views and engagement that followed each news story, news stories were arguably one of the most effective outreach mechanisms used during the observed recruitment period. They are also one of the most difficult outreach mechanisms to implement, depending on either significant financial resources or existing interpersonal relationships with a news station or anchor. The MAGENTA study benefited from existing relationships between our patient advocate partners and local news anchors. If traditional media outreach such as this can be obtained, it can clearly be instrumental in meeting recruitment goals; however, it is unrealistic to count on it as a primary outreach mechanism. In addition, outreach that is geographically focused, such as news stories released over a specific network, will ultimately be limited to the demographic served by that network. This was certainly the case for the WCCO story, which is discussed in further detail in the following sections.

The WCCO televised story was arguably the most successful individual recruitment effort [44]. This story featured a local news anchor with a family history of breast and ovarian cancer named Kim Johnson. Johnson is an established household name for many of the communities served by the WCCO and has spoken publicly about ovarian cancer in the past. It is possible that this story gained the traction it did for the same reasons that web-based information seekers are more likely to use familiar sources—if they can recognize the name, they are more likely to trust it [49]. Comparing these efforts with the enrollment activity following paid advertisements, it appears that although paid advertisements have an impact, collaboration with patient advocacy groups is also important for reaching a target audience. By leveraging existing social networks over social media through patient advocacy groups, Facebook could offer more cost-saving opportunities for research recruitment, particularly for large-scale studies such as MAGENTA. Considering these opportunities, as the average Facebook user continues to age [50], Facebook is likely to become an increasingly favorable venue for recruiting adults for research. A similar evolution in the average user is also observable across other social media platforms.

### Web-Based Behavioral Advertising Supports Message Delivery—But Not to Everyone

OBA made it easy to target information about the study to specific age groups, regions, and expressed and inferred interests. For example, we were able to target people who met the age and regional criteria and who had expressed an interest in various ovarian and breast cancer–related initiatives. Women who were aged ≥65 years had the best response rate when compared with other age groups, with approximately 43.95% (15,124/34,410) of reaches translating to engagement. This response rate suggests that although individuals aged ≥65 years make up a smaller percentage of web-based social media users, they are arguably more responsive to the content they see on social media than younger demographics. Their response rates could potentially be leveraged with a different message. Rather than encouraging them to enroll themselves, future advertisements might implore them to encourage their family members to learn more about the MAGENTA study.

Facebook and other social media platforms certainly present several opportunities for researchers; however, privacy concerns and worries over the use of OBA data make it clear that the drivers of Facebook do not always share the same values as the drivers of research. Paid advertising presents unique opportunities to target specific groups of people; however, unpaid posts published across existing web-based social networks are arguably preferable from an ethical standpoint, particularly with regard to recent data breaches on Facebook and concerns about how social media platforms such as Facebook use and monetize OBA data [51]. Data privacy issues such as these affect consumer trust and may deter users from previously trusted social media platforms, such as Facebook. Importantly, when unpaid posts come from existing social media profiles, such as a patient advocacy Facebook page with an established following, it is likely to function better than a sponsored advertisement, in large part because of this trust factor. When a message comes from a trusted source, patients are more likely to feel comfortable engaging with it. This requires research teams to build relationships with patient advocacy groups, specifically with those that include a following that meets the intended study eligibility criteria. In the absence of this invaluable resource, paid advertising may offer an effective alternative.

Most MAGENTA participants were White-identifying individuals. This may have been partly because of the geographic locations that recruitment bursts originated from; for example, the Minnesota burst increased enrollment from a region comprising >80% non-Hispanic White individuals. Black and indigenous people of color are chronically underrepresented in clinical research settings [52]. This trend is partly explained by ineffective recruitment mechanisms [53]. The relatively homogenous sample recruited by the MAGENTA study poses a deficit for research, leaving underrepresented communities less likely to clinically benefit from research findings [52]. This problem is not unique to MAGENTA and is not something that social media recruitment alone can resolve.

Prior work suggests that different groups have different response rates where research is concerned, meaning that targeted marketing, even over social media, is likely to leave certain groups underrepresented [34]. Current recommendations highlight the importance of allowing the target population to inform platform choices [26]. Other social media platforms with sufficient Black and indigenous people of color representation should be explored for recruitment opportunities. Future research should assess the effectiveness of targeted recruitment across varying social media platforms for the purposes of reaching underrepresented populations and exploring alternative delivery models to improve access to genetic testing for Black and indigenous people of color communities.

A drop-off was observed from the initial engagement to enrollment and randomization (Table 2). The drop-off may be explained by a normal study drop-off at each stage; however, it may also be the result of the complex web-based enrollment protocol used. Participants who learned about the study were referred to the study webpage, and there, they were several clicks away from the eligibility questionnaire (Figure 1). Eligible individuals then had to note the messaging at the end of the questionnaire that told them to check their email inbox for an email containing the next steps and ensure that any auto-filtering system they had turned on in their email inbox did not filter the REDCap email directly into their trash or spam box. This issue came up during initial system usability testing and was addressed by adding additional messages at the end of the questionnaire, which prompted people to check their email inboxes. It is possible that some of the drop-off rates between the completion of the questionnaire and providing signed consent may be because of lost emails.

### Limitations

There were several limitations to this study. One of the most prominent issues was the varying definitions of *reach* and *engagement* across different web-based platforms. Although Facebook differentiated these variables, REDCap did not, making it difficult to accurately compare numbers across the various platforms included in the web-based communication system. This also made it difficult to determine whether a particular effort was successful or whether the participant finally took action after seeing information about the study for the third or fourth time, a potential trend supported by marketing research that indicates that repeated exposure is required for action [54]. Similarly, Facebook does not currently have a way of tracking website conversions through unpaid posts or a public-facing means of tracking the demographics of engaged users or platforms from which they access content; thus, this information was not collected for unpaid published materials.

The platform itself also has a limitation. First, Facebook, similar to other social media platforms, is a rapidly evolving tool that uses internal user analytics to make changes to its terms and use agreements. This includes routine revisions of advertising platforms. For MAGENTA, this meant that some of the initial targeting variables and content used toward the beginning of the observed recruitment period were no longer available as the outreach continued. Although this is an issue that all social media platforms are likely to face, there are other reasons that researchers should carefully consider their options in social media platforms when choosing one for recruitment outreach.

Platform selection appears to be one of the most important factors in conducting social media research. The most popular social media platforms currently used for research recruitment are Facebook, Twitter, and Instagram. Each of these platforms has different user demographic profiles, with social media preferences varying by race, ethnicity, and age. Facebook is increasingly becoming a platform that is appropriate for reaching middle-aged and older adults as the average Facebook user ages [18]. Instagram and Twitter, on the other hand, may be better options for reaching younger populations, given that the average Instagram and Twitter user is aged <35 years [18]. The average Twitter user, for example, is a young, affluent, college-educated male of color [18]. Certain racial-ethnic groups also tend toward other preferred social media platforms. For example, the most popular social media platform among Koreans is called KakaoTalk [55]. It is important to choose a social media platform populated by members of the intended population. This requires an understanding of the social media habits of the target population. It is also critical to understand that any social media platform will be subject to sampling bias if used to recruit research participants. Not only will recruitment activities be subject to the bias present on the specific platform but also be subject to the bias that results from internet-based recruitment efforts; that is, the resulting study population will largely be made up of individuals who use the internet, a potential marker of eHealth literacy and technology literacy. Regardless of the research goals, the target population should inform the social media platform choice.

## Abbreviations

CA: California
MAGENTA: Making Genetic Testing Accessible
OBA: online behavioral advertising
REDCap: Research Electronic Data Capture
SU2C: Stand Up To Cancer
WA: Washington
